# Antibacterial and anti-biofilm activity of diarylureas against *Enterococcus faecium* by suppressing the gene expression of peptidoglycan hydrolases and adherence

**DOI:** 10.3389/fmicb.2022.1071255

**Published:** 2022-12-15

**Authors:** Yunfeng Xie, Lei Wang, Yang Yang, Liang Zha, Jiazhen Zhang, Kuanrong Rong, Wenjian Tang, Jing Zhang

**Affiliations:** ^1^School of Medicine, Anhui University of Science and Technology, Huainan, China; ^2^Anhui Prevention and Treatment Center for Occupational Disease, Anhui No. 2 Provincial People's Hospital, Hefei, China; ^3^School of Pharmacy, Anhui Medical University, Hefei, China

**Keywords:** *Enterococcus faecium*, diarylurea, biofilm, peptidoglycan hydrolase, quorum sensing, SagA

## Abstract

*Enterococcus faecium* (*E. faecium*) is a clinical multidrug-resistant pathogen causing life-threatening infection, which makes it important to discover antibacterial agents with novel scaffolds and unique mechanism. In this study, the diarylurea scaffold was found to have potent antibacterial effect on *E. faecium*. Diarylurea **ZJ-2** with benign drug-like property exhibited potent antibacterial and anti-biofilm activity through inhibiting the genes expression of NlpC/p60 hydrolase-secreted antigen A (*sagA*) and autolysins (*atlA*), down-regulating the expression of biofilm adherence related genes aggregation substance (*agg*), enterococcal surface protein (*esp*) against *E. faecium*. Moreover, **ZJ-2** can be docked into SagA to inhibit daughter cell separation. In a mouse model of abdominal infection, **ZJ-2** decreased the bacterial load and the level of IL-6 and TNF-α in a time-dependent manner. Overall, these findings indicated that diarylurea **ZJ-2** has the potential to be developed as a therapeutic agent to treat drug-resistant enterococci and biofilm-related infections.

## Introduction

Enterococci are common causes of nosocomial infection, and multidrug-resistant species can cause biofilm-associated infections, including in the urinary tract, wounds, dysbiotic gastrointestinal tract (Landete et al., 2018). *E. faecalis* is one of the most important clinical enterococci, which cause the majority of the infections in hospitals and effectively “escape” the effect of antibacterial agents (Abat et al., 2016; Santajit and Indrawattana, 2016; Ch'ng et al., 2019). Drug-resistant biofilm formation makes *E. feacium* difficult to completely eradicate by first-line antibiotics (Mohammad et al., 2017; Ciofu et al., 2022). Enterococci can acquire resistance to quinolones, macrolides, tetracyclines and glycopeptides, etc. (Conwell et al., 2021). Therefore, it is significant to discover anti*-E. feacium* agents with novel scaffolds and unique mechanism.

The thick cell wall (40 nm) of enterococci is mainly composed of tough and resilient peptidoglycan (PG), which makes it difficult to destroy (Culp et al., 2020). PG functions as a scaffold for the attachment of proteins and other polymers essential for cell growth and division, microbial pathogenesis, and antibiotic resistance (Hernández-Rocamora et al., 2018; Moynihan et al., 2019). Hydrolases and synthases can modulate invasion of prey cells, cell shape, innate immune detection, and intercellular communication, and inhibit or stop bacterial growth and daughter cell separation at very low level (Rangan et al., 2016; Griffin et al., 2021). The secreted antigen A (SagA) and autolysin (AtlA) are the major NlpC/p60 PG peptidoglycan hydrolases of *E. faecium* involved in cell division and cellular autolysis (Pedicord et al., 2016; Stinemetz et al., 2017). SagA breaks down bacterial cell walls and is the major protein secreted during biofilm formation in *E. faecium* (Stinemetz et al., 2017; Espinosa et al., 2020). AtlA is responsible for eDNA release in *E. faecium* biofilms and plays an important role in the division of daughter cells following replication through its autolytic activity (Stinemetz et al., 2017). The component changes of cell wall in the biofilm may help to understand antibacterial mechanism of urea scaffolds.

Biofilm formation and dispersal are highly regulated by quorum sensing (QS) at the genetic level (Lu et al., 2020; Attallah et al., 2022). Adherence-related virulence factors include *esp*-encoded surface protein, *agg*-encoded aggregation substance (Soares et al., 2014; Spiegelman et al., 2022). Arylurea scaffolds displayed potent anti-bacterial activity by eradicating biofilms, which would promote us to study the relationship between anti-biofilm effect and QS regulation (Abdel-Rahman and Morsy, 2007; Elekhnawy et al., 2022; Zha et al., 2022).

In this work, a series of diphenylureas were synthesized and found as antibacterial agents against drug-resistant *E. faecium* (Supplementary Figure S1), amongst them, diphenylurea **ZJ-2** showed the most potent activity. **ZJ-2** was used to study the anti-biofilm mechanism on the regulation of peptidoglycan hydrolase genes and adherence-related genes. Diarylurea scaffold may be developed as a novel therapeutic agent to treat drug-resistant enterococci and biofilm-related infections.

## Materials and methods

### General

All chemicals, reagents and solvents were purchased from commercial sources and used without further purification. Reactions were checked by thin-layer chromatography on silica gel plates (Qingdao Marine Chemical Factory, GF_254_); spots were visualized by UV at 254 nm. Melting points are determined and are not corrected on a XT4MP apparatus (Taike Corp., Beijing, China). ^1^H NMR and ^13^C NMR spectra were recorded on Bruker AV-400 or AV-500 MHz instruments using DMSO-*d*_6_ as solvent. Chemical shifts are reported in parts per million (*δ*) downfield from the signal of TMS as internal standards. Coupling constants are reported in Hz. The multiplicity is defined by *s* (singlet), *d* (doublet), *t* (triplet), or *m* (multiplet). High resolution mass spectra (HRMS) were obtained on an Agilent 1260-6221 TOF mass spectrometry.

### Bacterial strains and media

The reference strains were *E. coli* ATCC25922, *P. aeruginosa* ATCC 27853 and *S. aureus* ATCC25923. 14 Gram-negative bacteria, 36 Gram-positive bacteria and 7 *Candida strains* were isolated from the urine, blood and pus of patients. Antibiotic susceptibility testing was performed by using Vitek2 Auto systems. Cation-adjusted Mueller-Hinton broth (CAMHB), crystal violet, Trypsin–EDTA, Tryptic soy broth (TSB), Tryptic soy agar (TSA), phosphate-buffered saline (PBS), Dulbecco’s modified Eagle’s medium (DMEM), fetal bovine serum (FBS), and 96-well plates were all purchased from commercial vendors (BD, Cat.). XTT sodium salt (shanghai yuanye Bio-Technology Co., Ltd), Menadione (shanghai macklin biochemical Co., Ltd).

### Minimum inhibitory concentrations assay

MIC assay was determined using the MH broth microdilution procedure described by the Clinical and Laboratory Standards Institute (CLSI). MIC was the lowest concentration of tested compounds in 96-well micro-test plates. Tested compounds were dissolved in an aqueous solution containing 10–20% DMSO to make the concentration of stock solutions be 20 mmol/L. Then 1 ml of the stock solution was added to 9 ml sterile water and diluted to 200 μmol/L. The wells of columns 1–12 of standard 96-well microdilution plates were filled with 100 μl Mueller-Hinton Broth and compounds were dispensed into the highest concentration 200 μmol/L in column 1. Two-fold serial dilutions were then made in the plates from columns 2–10. The initial bacterial inocula was 10^8^ CFU/ml. The final concentration of test compounds ranged from 0.39 to 200 μmol/L and bacterial inocula was diluted to 10^6^–10^7^ CFU/ml. Plates were incubated at 37°C for 20–24 h, after incubation, microplates were removed from the incubator. MIC values can be determined based on the use of a spectrophotometer (Bio-Tek Epoch-2, United States) method. MIC values were read and recorded as the lowest concentration of compounds that inhibited the visible growth of the organism. All assays were done with triplicate independent inocula (Weinstein and Lewis, 2020).

### Cell viability

Cell viability was performed against the human hepatocellular liver carcinoma cells (HepG-2) using the MTT assay. HepG-2 cells were grown in DMEM containing 10% fetal calf serum, 100 units per mL penicillin and 100 mg/ml streptomycin at 37°C in a 5% CO_2_ incubator. HepG-2 cells were seeded at 1 × 10^4^ cells per well in 96-well micro-test plates. After 24 h of culture, the cells were treated with different concentrations of tested compounds. After 24 h, 20 ml of 0.5 mg/ml MTT reagent was added to the cells and incubated for 4 h. After incubation, the liquid in the well was discarded, and 150 ml of DMSO was added to dissolve the formazan. The absorbance value (OD_570_) was measured at 570 nm. The cell percentage survival rate was calculated by setting the density of formazan formed in the blank group to 100% viability as a control. Cell viability (%) = compound (OD_570_)/blank (OD_570_) × 100%. Each compound was tested in triplicate.

### Docking simulations

The CDOCKER molecular docking module was performed using Discovery Studio 2018 (V18.1.0.17334) software to explore the ability of **ZJ-2** targeting SagA (PDB code: 2OXN). Before docking simulation, all water molecules were deleted and ligand **ZJ-2** was prepared with standard protocol using Discovery Studio 2018. The protein structure was carefully treated in several steps including residue repairing, protonation, and partial charges assignment in CHARMm force field. The target enzyme was prepared with Prepare Protein of DS to ensure the integrity of target. The ligand was processed by Full Minimization of the Small Molecular in DS. The binding pocket was defined by the crystal ligand. **ZJ-2** was inserted into the substrate binding pocket of SagA to replace RXV. All docking calculations were performed using default settings.

### Growth inhibition assay

The assay was used to study antimicrobial activity and can determine the bactericidal or bacteriostatic activity of an agent over time. MIC plate was prepared as previously described giving a final cell density 5 × 10^5^ CFU/ml, **ZJ-2** was incubated with *E. faecium* E3101 at 37°C in a concentration range of 1/2MIC, MIC, 2MIC, and 4MIC. The effect of four concentrations was quantified after 0, 4, 8, 12, 16, 20, and 24 h incubation at 37°C. At each time point, an aliquot (100 μl) was pipetted and measured for the OD_600_ nm. The experiment was carried out in three biologically independent assays and each sample was tested in triplicate.

### Broth dilution serial passage resistance induction studies

The resistance induction (22-day generational passage) assay was conducted for **ZJ-2** and linezolid. The clinical strain of *E. faecium* E3101 was exposed to sub-MIC of **ZJ-2** and incubated at 37°C overnight to determine viable colony count. Linezolid for sustained passages and the new values of the tested compound for each passage of strains were determined. The bacteria from sub-MIC concentration of tested compound (1/2MIC) were diluted for the next MIC experiment. After 24 h of incubation, the bacteria from sub-MIC concentration of compound (1/2MIC) were diluted for the next MIC experiment. This process was repeated for 22 passages. The new MIC values of compound against each passage of strains were determined.

### Biofilm assay

*E. faecium* E3101 was cultured in MH broth at 37°C overnight. Then 100 μl MH broth was added to the wells of 96-well plates, the first and last line, as the controls. Plates were plated in duplicate and incubated at 37°C for 48 h to allow for biofilm formation and **ZJ-2** was tested as a 12-point dose–response from 0.049 to 100 μmol/L. Linezolid and **ZJ-2** were serially two-fold diluted in MH broth, After 24 h incubation, the biofilm-containing plates were washed three times with 200 μl per well of saline solution to remove the planktonic cells but leaving the biofilm uninterrupted. Then 100 μl compound solution was transferred into the washed biofilm containing plates and incubated at 37°C for a further 24 h. After incubation, plates were washed three times, then fixed with 99% methanol for 15 min. Then 100 μl per well 0.1% crystal violet (CV) stain was added for 20 min, 150 μl per well of methanol was added to indicate minimum biofilm eradication concentration (MBEC), which was determined at OD_595_.

### XTT reduction assay

Metabolic activity of biofilm was determined by using XTT (2,3-bis(2-methoxy-4-nitro-5-sulfophenyl)-2*H*-tetrazolium-5carboxanilide) reduction assay. The formed biofilms of *E. feacium* were washed with sterile PBS to remove non-adherent cells in each well. Then, incubated with containing different concentrations of **ZJ-2** (MIC, 2MIC, and 3MIC) at 37°C for an additional 24 h. At the end of the incubation period, the biofilms were washed with PBS, and the metabolic activity was quantified using the XTT. The sodium salt of XTT was dissolved in PBS at a final concentration of 1 mg/ml. Menadione was dissolved in acetone to 1 mmol/L. 100 μl PBS was added in each well, 13.5 μl of fresh XTT/menadione mixture (12.5: 1) was mixed in each well, incubated at 37°C for 3 h, then absorbance was read in three replicates at 490 nm (Pourkhosravani et al., 2021).

### Electron microscope

The *E. faecium* E3101 suspension was adjusted to 0.5 MacFarland units to give 5 × 10^7^ CFU/ml in MH broth, and treated for 1 h with **ZJ-2** at 0, 1/4MIC, 1/2MIC, respectively, and further cultured for 24 h at 37°C. After exposure, the treated and untreated cells were collected at 8000 g for 5 min, and rinsed with 0.1 M PBS. Then cells were fixed with 2.5% (v/v) glutaraldehyde for 16 h at 4°C and further dehydrated through a graded series of alcohol (30, 50, 70, 90, and 100%) for 15 min. After serial dehydration, CM-10 TEM analysis was used to examine fixed cells at 80 kV operating voltage. The SEM model used is the S4800 at 3.0 kV volt.

### *In vivo* assay

Animals: SPF female C57BL/6 J mice (aged 2–3 weeks; 18–20 g) were purchased from Henan Skebesi Biotechnology Co. Ltd.; Mice were maintained with SPF food and water for 1 week. The animal room is 20–26°C; warm Humidity is 40–70%; 12 h of light and darkness alternate; normal feeding before animal experiments. Mice were divided into five groups randomly: **ZJ-2** group (5 mg/kg), linezolid group (5 mg/kg), vancomycin group (5 mg/kg), bacteria control group and blank group, and 5 × 3 mice in each group. Except for blank group, each was intraperitoneally injected with 100 μl *E. faecium* E3101 suspension (2 × 10^6^ CFU/ml), after 2 h, subcutaneous injections were administered. The mice were euthanized 6, 12, and 48 h after infection, mice blood and spleen were harvested to count the viable bacteria. The level of IL-6 and TNF-α in serum was measured by ELISA assay, and liver tissue sections were taken for 48 h for HE staining.

### RNA extraction and qRT-PCR

The expression level of *E. faecium* genes were determined by qRT-PCR. The primers used for qRT-PCR are listed in Table 1. Total RNA was extracted from the *E. faecium* E3101 treated with **ZJ-2** (late exponential phase, 24 h) using the TRIzol method for RNA extraction and converted to cDNA and using SuperScript™ III First-Strand Synthesis SuperMix for qRT-PCR (Invitrogen, United States), and quantified with a LightCycler96 (Roche, Switzerland). The quantitative PCR cycle threshold (CT) results were analyzed using the comparative CT method (2^-ΔΔCT^ method) with some modifications. All kits were used according to the manufacturers’ instructions. Specific primers are listed in Supplementary Table S1. Amplification was performed in a gradient thermal cycler (Bio-Rad, Hercules, United States). All samples were analyzed in triplicate.

**Table 1:** MIC and antimicrobial susceptibility profiles of strains in this study.

Strain	MIC (μmol/L)							Number of resistance isolates						
	n	ZJ-1	ZJ-2	ZJ-3	ZJ-5	ZJ-8	ZJ-10	ERY	H-GEN	LVX	LZD	PEN	VAN	OXA
**Gram-Positive (resistant strains)**														
*S. aureus*	6	>200	0.78–1.56	3.13	>200	3.13	1.56–3.13	3	2	1	5	0	1	2
*E. faecium*	9	>200	0.78–1.56	6.25–12.5	>200	6.25–12.5	6.25–12.5	4	3	2	4	1	5	3
*E. faecalis*	5	>200	1.56–6.25	12.5	>200	12.5–25	12.5	1	1	0	2	2	2	1
*E. aviue*	1	>200	3.13	25	>200	25	25	0	0	0	1	0	1	0
*S. epidermidis*	4	>200	3.13–6.25	3.13–6.25	>200	6.25–12.5	6.25–12.5	0	1	1	2	1	2	3
*S. haemolyticus*	5	>200	1.56–3.13	3.13–6.25	>200	1.56–3.13	1.56--3.13	0	1	1	1	0	3	1
MRSA	6	>200	1.56–6.25	3.13–6.25	>200	6.25–12.5	6.25–12.5	2	3	2	4	2	2	4
**Gram-negative**														
*P. aeruginosa*	1	>200	>200	>200	>200	>200	>200							
*A. baumannii*	2	>200	>200	>200	>200	>200	>200							
*K. pneumoniae*	5	>200	>200	>200	>200	>200	>200							
*E. coli*	6	>200	>200	>200	>200	>200	>200							
**Fungus**														
*C. albicans*	4	>200	>200	>200	>200	>200	>200							
*C. neoformans*	3	>200	>200	>200	>200	>200	>200							

*S. aureus*: *Staphylococus aureus*; *E. faecium*: *Enterococcus faecium*; *E. faecalis*: *Enterococcus faecalis*; *E. aviue*: *Enterococcus aviue*; *S. haemolyticus*: *Staphylococus haemoly*; *P. aeruginosa*: *Pseudomonas aeruginos*; *A. baumannii*: *Acinetobacter baumannii*; *K. pneumoniae*: *Klebsiella penumoniae*; *E. coli*: *Escherichia coli*; *C. albicans*: *Candida albicans*; *C. neoformans*: *Candida neoformans*; MRSA: Methicillin-resistance staphylococcus aureus. ERY: Erythromycin; H-GEN: High-Gentamicin; LVX: Levofloxaci; LZD: Linezoild; PEN: Penicillin; VAN: Vancomycin; OXA: Oxacilliin.

### Statistical analysis

All statistical analyses were performed using SPSS software (SPSS 13.0 for Windows). The data are presented as the mean ± SD (*n* = 3). Differences between means were evaluated by Student’s *t*-test and defined as significant at *p* ≤ 0.05.

## Results

### Chemistry

Substituted arylamine **SM-1** was transferred into aryl isocyanate R–NCO (**1**) by triphosgene in basic condition, then substituted arylamine **SM-2** was added into the reaction solution to give the diarylurea products **ZJ-1**–**ZJ-12** (Supplementary Figure S1). Their structures were elucidated by spectroscopic methods including by ^1^H NMR, ^13^C NMR spectra and HRMS.

**Figure 1:**
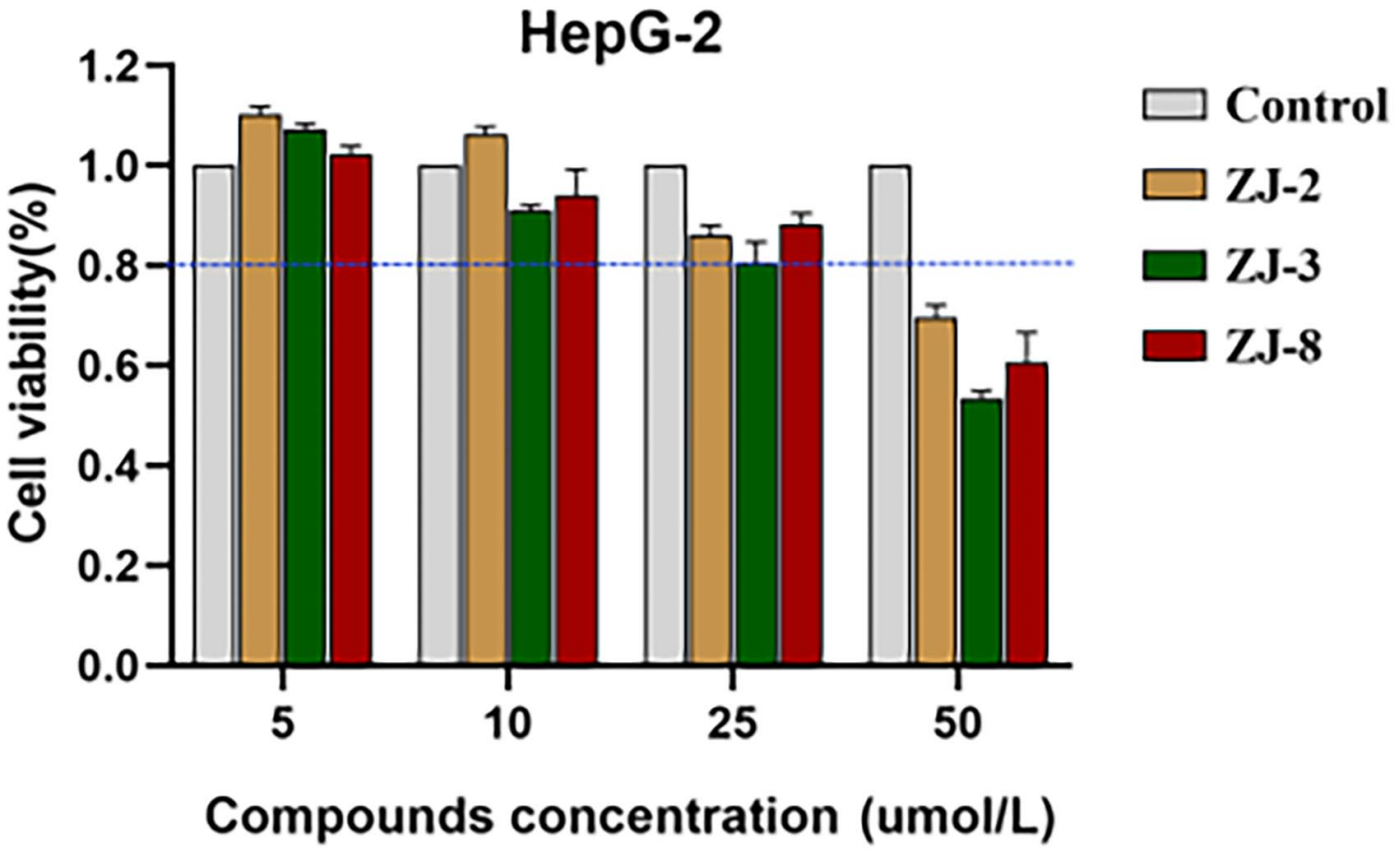
Cell viability assay of diarylureas to HepG-2 cells.

Twelve diarylureas ZJ-1–ZJ-12 were synthesized and screened for their antibacterial activity (Figure 1; Supplementary Figure S1; Scheme S1). SAR results showed: (i) the position of aryl ring affected the anti-bacterial activity, *p*- > *m*- > *o*-, ZJ-2 > ZJ-3 > ZJ-4, ZJ-5 > ZJ-6, ZJ-7; (ii) the substituent of aryl ring affected the anti-bacterial activity, -CF_3_, 3,4-diF, -F had better activity, amongst them, compounds ZJ-2, ZJ-3, and ZJ-8 with *p*-CF_3_ at phenyl ring exhibited potent anti-bacterial activity (MICs, 0.19 and 0.39, 0.39, and 0.39, 0.78, and 1.56 μmol/L, for SA and MRSA, respectively).

### Antibacterial activity

Twelve diphenylureas were evaluated for their antibacterial activity, amongst them, some compounds showed narrow-spectrum antibacterial activity against Gram-positive bacteria (Supplementary Table S2). Further, compounds ZJ-2, ZJ-3, and ZJ-8 showed consistent antibacterial activity against 36 drug-resistant Gram-positive strains, which were resistant to three or more antimicrobial agents of erythromycin, high-Gentamicin, levofloxaci, linezoild, penicillin, vancomycin and oxacilliin, but they showed no antibacterial activity against 14 Gram-negative bacteria and 7 *Candida strains* (Table 1). ZJ-2 performed the best antibacterial activity against *S. aureus* and *E. faecium* with MIC value of 0.78–1.56 μmol/L (Table 1).

As shown in Table 2, nine clinical strains of *E. faecium* were used to evaluate the biofilm formation assays and MIC determination of compounds **ZJ-2**, **ZJ-3**, and **ZJ-8**. The results showed that *E. faecium* E3101 showed the best ability of biofilm formation, and **ZJ-2** had the best antibacterial effect against all clinical strains of *E. faecium*. Intriguingly, **ZJ-2** had almost the same antibacterial activity against drug-resistant *E. faecium* as that against *S. aureus* and MRSA (Supplementary Table S3).

**Table 2:** MIC and biofilm formation values for the clinical isolates *Enterococcus faecium*.

*E. faecium*	Biofilm formation	MIC (μmol/L)					
	(OD_595_)	ZJ-2	ZJ-3	ZJ-8	ZJ-10	VAN^a^	LZD^b^
E1101	0.87	0.78	1.56	3.125	25	0.78	1.56
E2205	0.67	1.56	0.78	3.125	12.5	0.39	0.78
E0516	0.78	1.56	3.125	6.25	50	0.78	1.56
E2203	0.56	1.56	3.125	12.5	12.5	0.78	1.56
E2123	0.66	0.78	1.56	25	25	0.39	0.78
E2016	0.79	0.78	3.125	25	12.5	0.39	0.78
E0512	0.91	1.56	1.56	1.56	12.5	0.78	1.56
E3101	1.01	0.78	1.56	6.25	12.5	0.78	1.56
E1105	0.88	1.56	3.125	25	25	0.39	0.78

a VAN: It is vancomycin HCl (Solarbio®, Lot. No. 1115F021).

b LZD: Linezolid.(Solarbio®, Lot. No. L7990).

### Cell viability evaluation

To evaluate cytotoxicity of diarylureas, the HepG-2 cells were treated with different concentrations of tested compounds (5.0, 10.0, 25.0, and 50.0 μmol/L), DMSO as a control, after 24 h, cell viability was measured using MTT method. As shown in Figure 1, **ZJ-2**, **ZJ-3**, and **ZJ-8** at the test concentrations (25.0 μmol/L) did not significantly reduce cell viability after exposing HepG-2 cells for 24 h. The cell survival rate of ZJ-2 at 50 μmol/L was about 70%, while that of ZJ-3 was only 50%. But the cell survival rate at 25 μmol/L was more than 80%, this concentration was more 50 times than that of the MIC, which implied that **ZJ-2**, **ZJ-3** and **ZJ-8** were low cytotoxicity. Therefore, **ZJ-2** can be considered a candidate drug for further investigation on the antibacterial mechanism of the diarylurea scaffold.

### Molecular docking study

**Figure 2:**
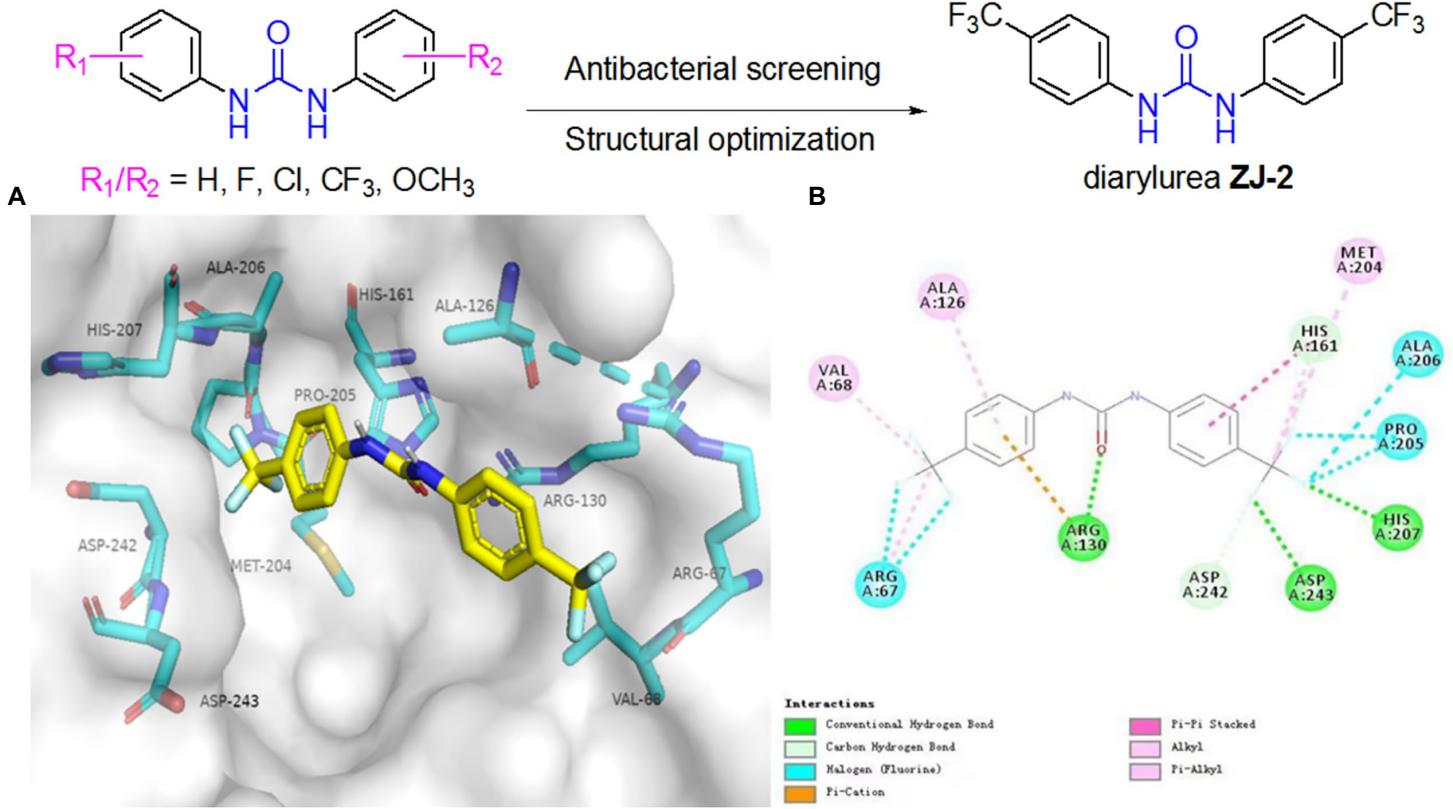
**(A)** Binding modes of **ZJ-2** with SagA (PDB code: 2OXN). **(B)** 2D mode of the interaction of **ZJ-2** with receptor SagA. Hydrogen bonds are shown in green, *p*–*p* interactions are shown in purple, halogen bonds are shown in blue and *p*-cation bond is shown in brown.

The docking results showed that **ZJ-2** inserted into the active pocket of SagA through extensive interactions, including π-π interaction interactions between the benzene ring and His161, three halogen bonds between the fluorine and Arg67, Pro205 and Ala206. hydrogen bond interactions are observed within the active site, two hydrogen bonds between the fluorine and Asp 243 and His 207, one hydrogen bond between the carbonyl oxygen and Arg130 (Figure 2). Altogether, the multiple interactions of the two aromatic moieties with the main pocket of the active-site gorge enlighten the high affinity of **ZJ-2** for SagA.

### Inhibition of bacterial growth

**Figure 3:**
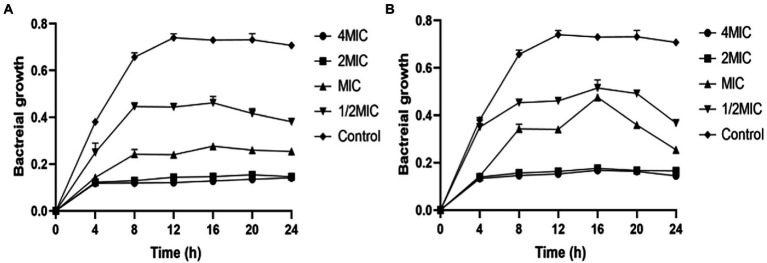
*E. faecium* E3101 growth inhibition curves. The curves showing the effect of **ZJ-2 (A)** and linezolid **(B)** on the growth of *E. faecium* E3101. Each OD point presented is the average values of three tests and all experiments are internally.

To further investigate the growth inhibition effects of **ZJ-2** and clinical antibiotic linezolid on *E. faecium* E3101, Growth inhibition assay was performed against *E. faecium* E3101. **ZJ-2** showed rapid growth inhibition (˃ 4 h), with a minimum inhibition concentration of 1.6 μmol/L (Figure 3A), which is better than linezolid (Figure 3B). The results showed that **ZJ-2** and linezolid were able to inhibit the growth of *E. faecium* E3101 effectively at the MIC or higher concentrations. Once the concentration dropped down to half of the MIC, they could slow down the growth rate, and the growth could be recovered after being incubated for a longer time.

### Anti-biofilm activity of ZJ-2

**Figure 4:**
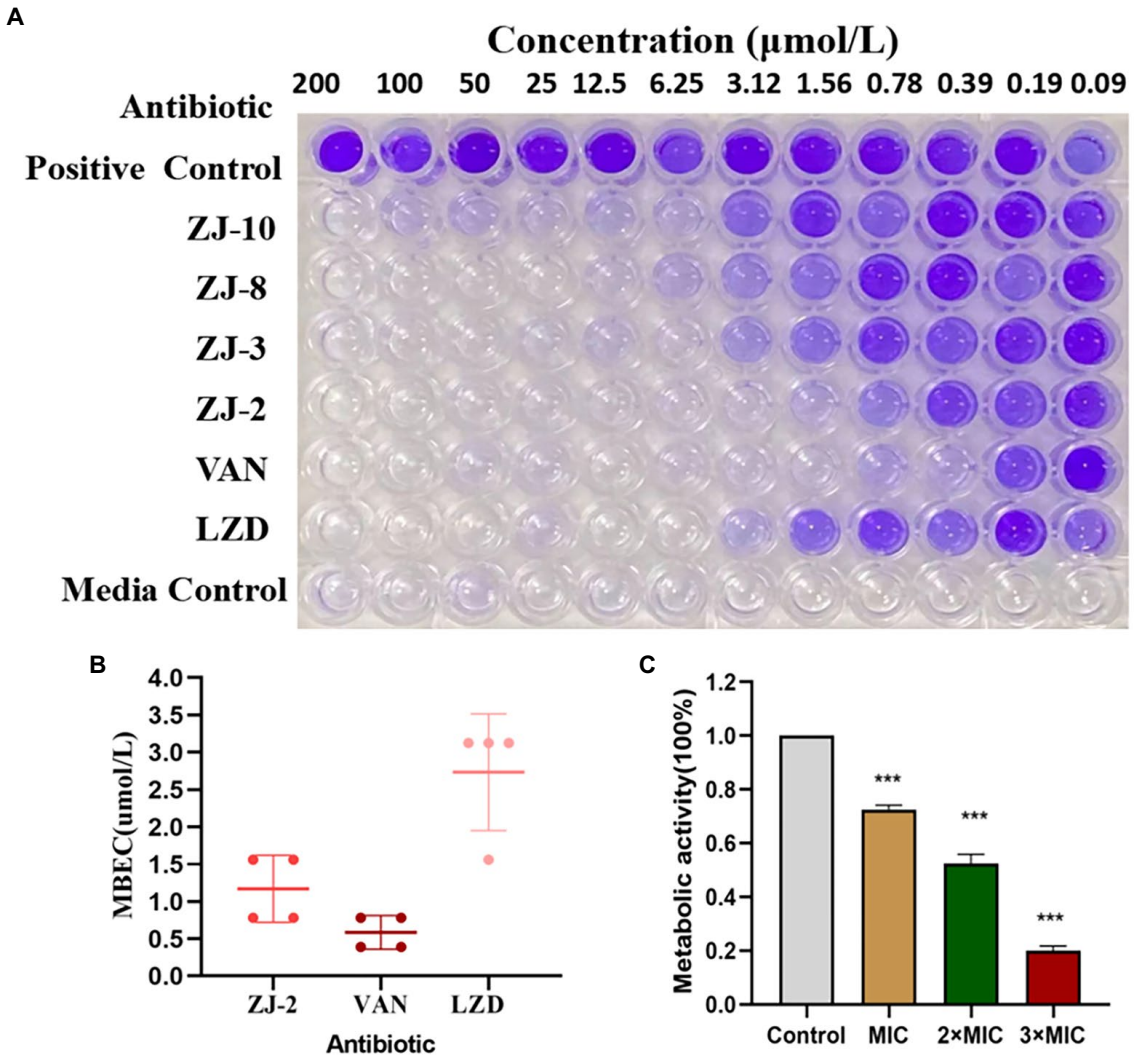
CV assay and XTT reduction assay. MBEC assessment of **ZJ-2** using crystal violet staining **(A)** to assess **(B)** E. *faecium* E3101 biofilm remaining after 24 h incubation with **ZJ-2**, vancomycin and linezolid (biofilm initially established by 48 h growth in TSB + 5% glucose). XTT reduction assay **(C)** to attenuate the metabolism of *E. feacium* biofilm After 24 h treatment of the established biofilms with **ZJ-2**. Each OD point presented is the average values of 4 tests. ***p < 0.01.

Forming antimicrobial-resistant biofilm is a significant phenotype of *E. feacium*, making it difficult to eradicate these bacteria by traditional antibiotics completely. To evaluate the remove biofilm efficiency of diarylurea, ZJ-2 was used to determine its eradication of established biofilm of *E. feacium E3101*. The CV assay showed that ZJ-2 inhibited biofilm formation of *E. faecium E3101* in a dose-dependent manner (Figure 4A), and exerted a minimum biofilm eradication concentration (MBEC) of 1.56–0.78 μmol/L, similar to its MIC, superior to linezolid against *E. faecium E3101* (Figure 4B). The XTT assay showed that after **ZJ-2** treatment with MIC, 2MIC and 3MIC, the metabolic activity of *E. feacium* E3101 was inhibited by 27.4, 49.8, and 79.7% in a dose-dependent manner, respectively, which revealed that the biomass and metabolic activity were consistent (Figure 4C).

### Broth dilution serial passage resistance induction studies

**Figure 5:**
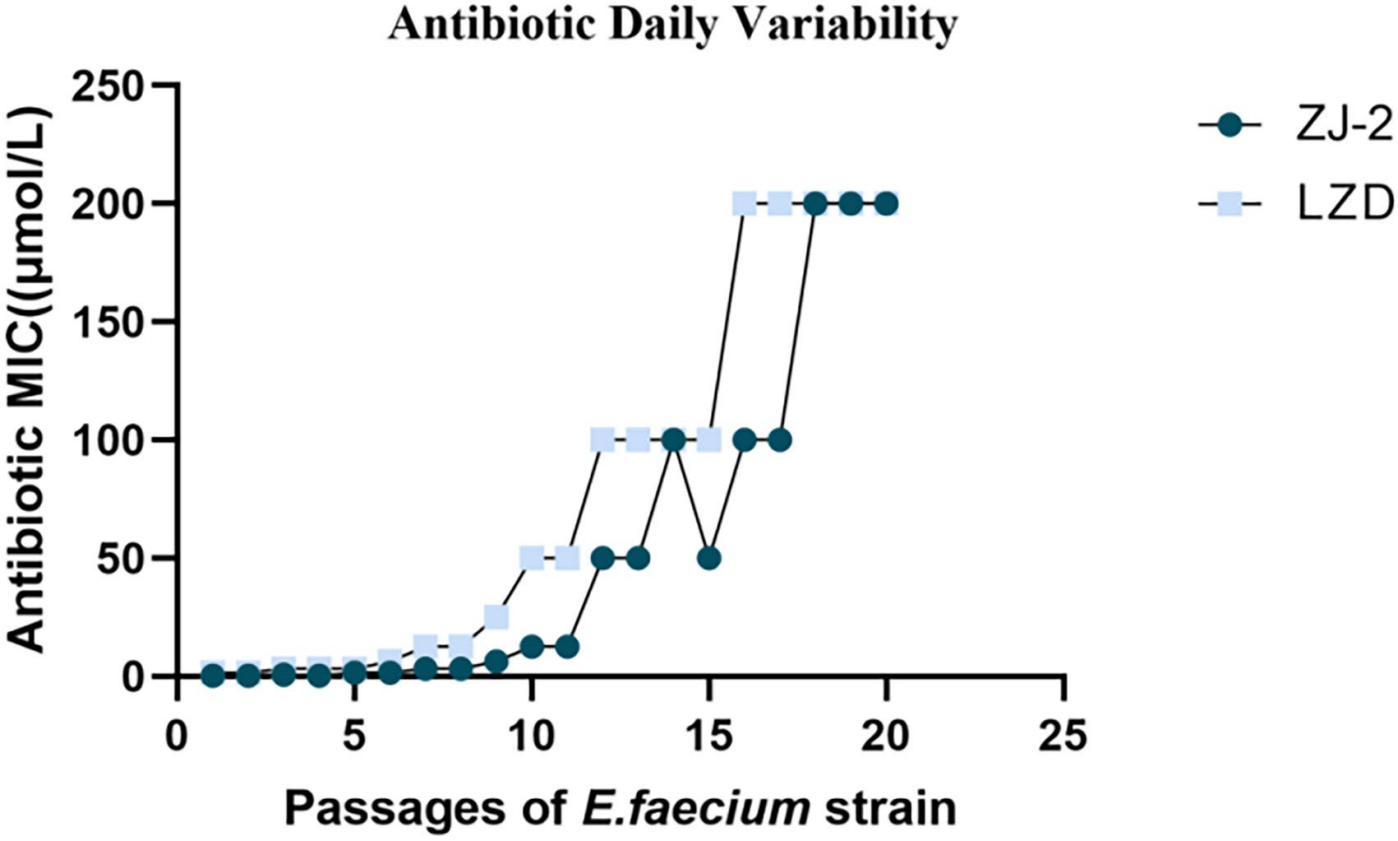
Broth dilution serial passage resistance induction studies. Average daily MIC during exposure of *E. faecium* E3101 to sub-MIC concentrations of linezolid over 22 days of bacterial growth.

To evaluate the efficacy of ZJ-2 for eluding acute anti-bacterial resistance mechanisms, we performed a stepwise, liquid culture resistance assay by consecutively passaging the *E. faecium* E3101 strain for generations over 22 consecutive days in the presence of serially diluted ZJ-2 and linezolid for comparison. At the end of each generation, MIC values were determined for test compounds, with the assumption that MIC values would increase over time if *E. faecium* E3101 was able to generate acute resistance. A plot of MIC values over time is presented in Figure 5. The MIC of ZJ-2 against *E. faecium* E3101 was induced to 64-fold at generation 14, and no longer changed after generation 18 to fold, while linezolid was induced to 128-fold at generation 10. These results suggested that ZJ-2 demonstrated a low propensity to induce resistance against *E. faecium* E3101.

### Electron microscope

**Figure 6:**
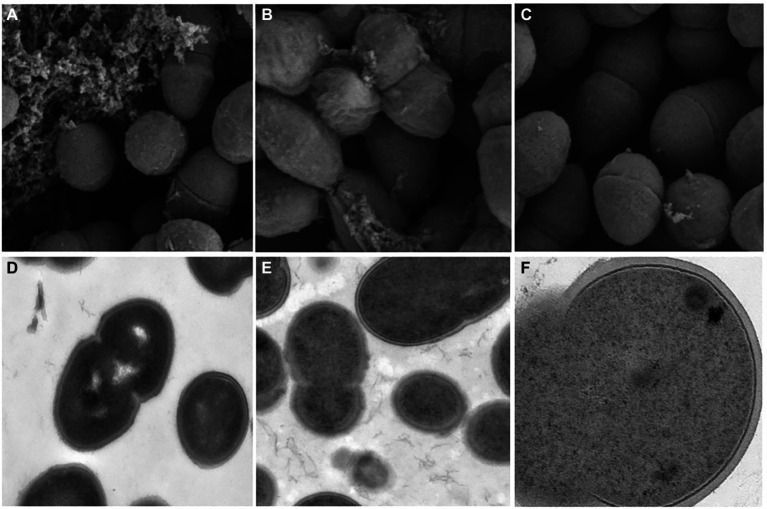
SEM images of *E. faecium* E3101 **(A–C)**, TEM images of *E. faecium* E3101 **(D–F)**. **(A,D)** represent untreated bacteria. **(B,E)** represent bacteria treated with **ZJ-2** at 1/4 MIC. **(C,F)** represent bacteria treated with **ZJ-2** at 1/2 MIC. SEM images’s view are 1.00 μm, TEM images’s view are 5.00 μm **(D,E)**, and TEM images’s view are 200 nm **(F)**.

The Scanning Electron Microscopy (SEM) and Transmission Electron Microscopy (TEM) analyses explicated that the cell wall of multidrug-resistant *E. faecium* was destroyed and the formation of biofilm was reduced at low concentration of ZJ-2 treatment. The SEM analysis showed the untreated biofilms in normal growth conditions (Figure 6A), after *E. faecium* E3101 was treated with ZJ-2 at 1/4MIC, 1/2MIC, the adhesion and covered area of the biofilm aggregation decreased (Figures 6B,C).

The results of TEM showed that the cell morphology changed when *E. faecium* E3101 was treated with **ZJ-2** at 1/2MIC and 1/4MIC. The untreated samples were bacteria-like with a smooth surface (Figure 6D), and the intracellular protoplasm was evenly distributed with high density (Figure 6E). After *E. faecium* E3101 was treated with 1/4MIC, cell membrane damage was observed. When exposed to 1/2MIC, the bacterial wall ruptured and the solute leaked (Figure 6F).

### ZJ-2 inhibits peptidoglycan hydrolase genes and biofilm adhesion-related genes expression

**Figure 7:**
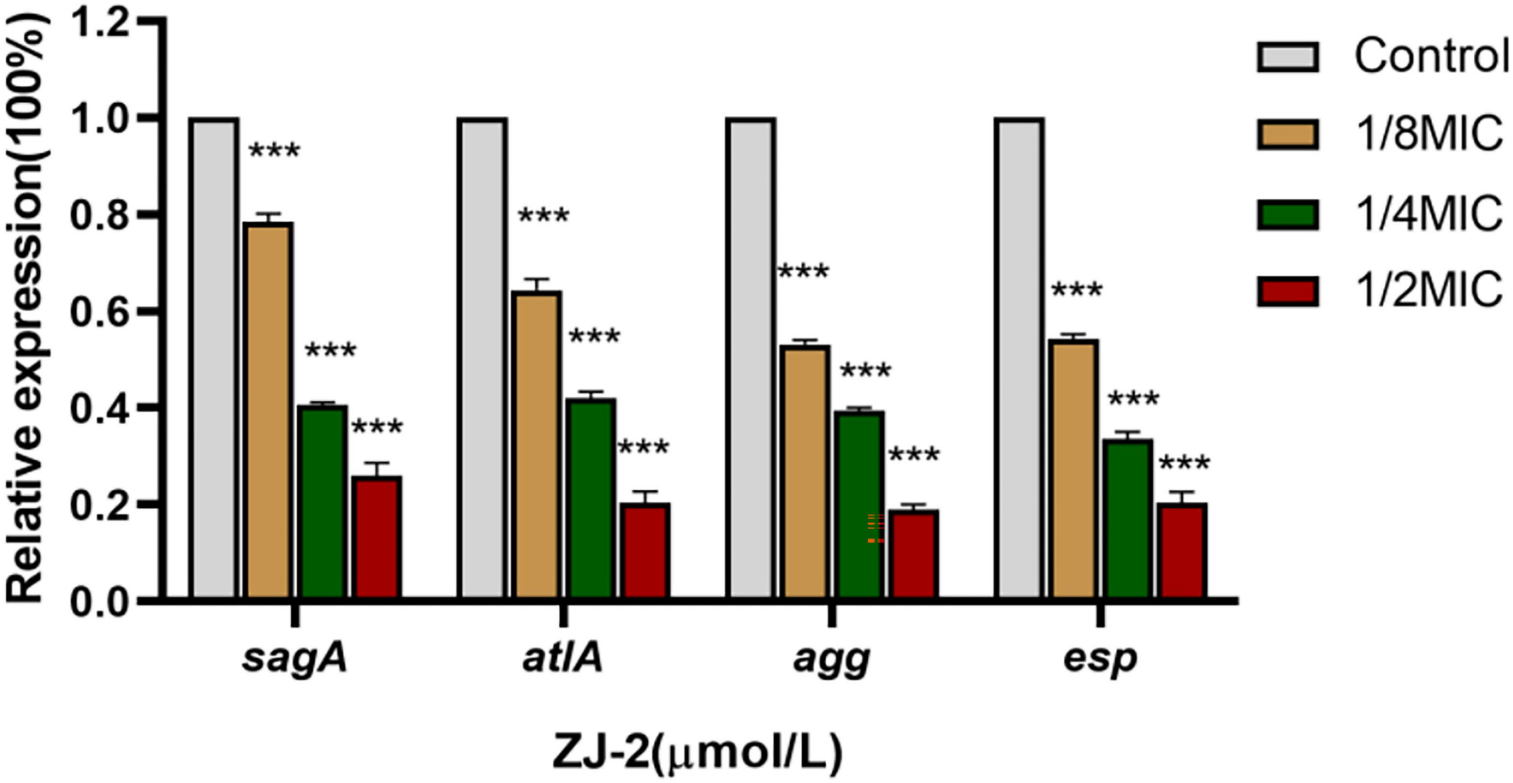
**ZJ-2** decreased the expression of the *sagA* and *atlA* genes and adherence-related genes *agg* and *esp* in E3101 (^***^p < 0.01).

The first step in biofilm formation is surface adhesion, in which, aggregation substance (Agg) and surface protein (Esp) play a major role in adhesion between *E. faecium* and renal tubular cells and enterocytes (Soares et al., 2014; Spiegelman et al., 2022). SagA and AtlA are the major PG hydrolases of *E. faecium* involved in cell division and cellular autolysis (Rangan et al., 2016; Stinemetz et al., 2017). The mRNA expression of *sagA*, *atlA, agg* and *esp* was decreased in a concentration-dependent manner after **ZJ-2** treatment in the degraded *E. faecium* E3101 cell wall (Figure 7). These results indicated that **ZJ-2** can break down bacterial cell walls and reduce the initial adhesion of biofilm by down-regulating the expression of PG hydrolase gene (*sagA* and *atlA*) and adherence-related genes (*agg* and *esp*) in a dose-dependent manner. Thus, Agg and Esp-deficient bacteria reduced initial attachment and reduced biofilm formation and virulence factors.

### Effect of ZJ-2 treatment on bacterial load in blood

**Figure 8:**
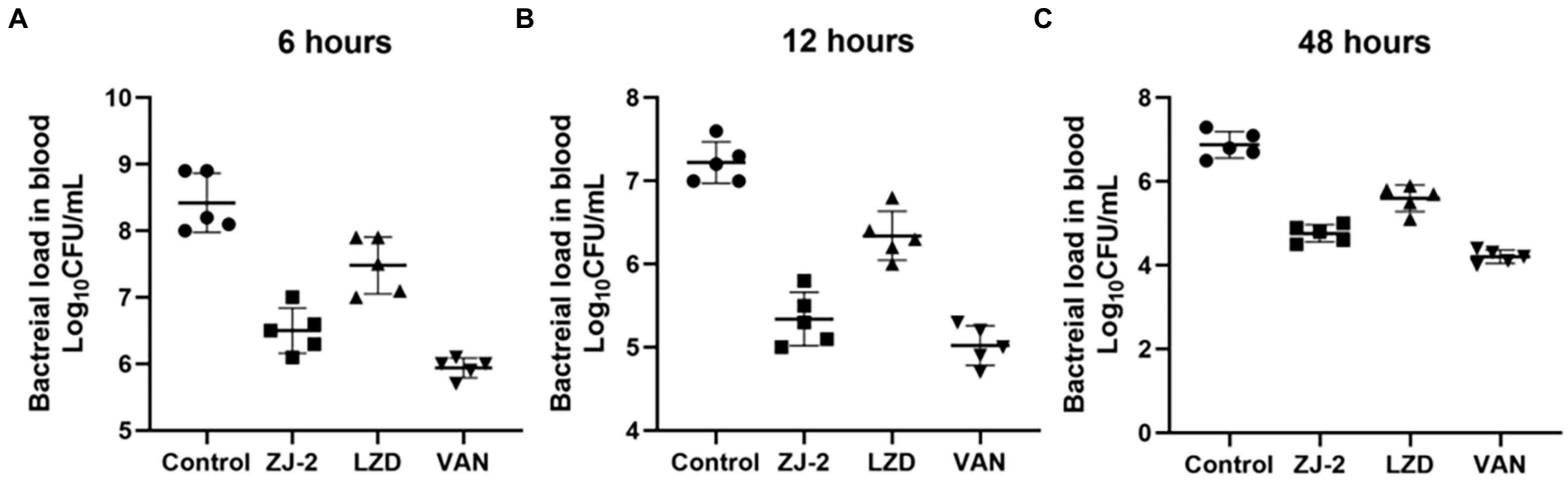
Effect of treatment with **ZJ-2** on bacterial load in the blood at 6 h **(A)**, 12 h **(B)**, and 48 h **(C)** against *E. faecium* E3101. Each symbol represents the viable bacterial counts determined in each animal, and the horizontal lines represent the average value for each group. Each OD point presented is the average values of 5 tests.

The antibacterial effect of ZJ-2 on *E. faecium* E3101 was evaluated in the mouse abdominal infection model. As shown in Figure 8A, the viable bacteria counts of the peritoneal fluid 6 h after inoculation in ZJ-2 group were significantly decreased compared with those in the LZD group, but not to those in the VAN group. There was no significant difference in the viable bacterial counts in each group. At 12 h (Figure 8B), the viable bacteria counts of the peritoneal fluid in ZJ-2, VAN, or LZD-treated groups were significantly decreased compared with those in the bacteria control group. Observation mice survived for 48 h without death in either ZJ-2, VAN or LZD compared to untreated animals. ZJ-2 could significantly reduce the bacterial load in the blood of mice, and the bacterial colony counts decreased by 4.51 to 5.04 log_10_ CFU/mL (Figure 8C). The efficacy of ZJ-2 was superior to that of LZD, which suggested that ZJ-2 could effectively reduce the *E. faecium* abdominal infection.

### Effect of ZJ-2 treatment on the levels of IL-6 and TNF-α

The effect of ZJ-2 on the Levels of IL-6 and TNF-α was observed after 12 h of the treatment. ELISA assay was conducted to determine the change levels of serum IL-6 and TNF-α and collect the total efficacy in 4 groups.

**Figure 9:**
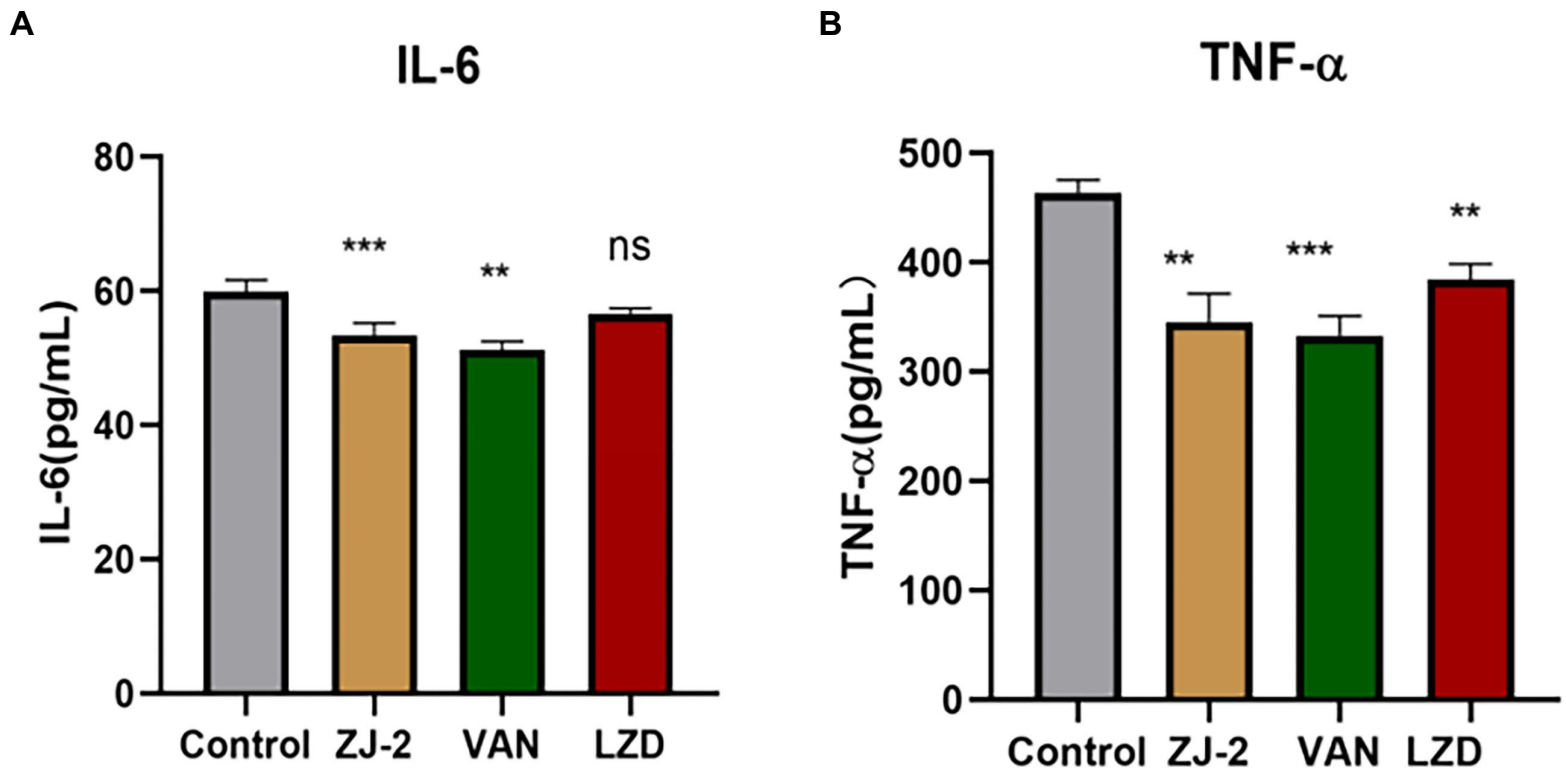
The IL-6 **(A)** and TNF-α **(B)** level in the serum at 12 h after *E. faecium* E3101 inoculation (*n* = 4). Values represent means ±SD; ^***^p < 0.01, ^**^p < 0.05.

Compared with bacteria control group, there was significant difference in serum IL-6 level for **ZJ-2**-treated group and vancomycin group, but there was no significant difference for linezolid group. Meanwhile, the serum TNF-α level was significantly different in treatment groups compared with control group, and **ZJ-2** showed better inhibition than linezolid (*p* < 0.01; Figure 9). These results indicated that **ZJ-2** may exert anti-bacterial activity through decreasing the levels of serum inflammatory factors caused by *E. faecium* E3101 infection.

### Effect of ZJ-2 on the pathological changes of liver tissue

**Figure 10:**
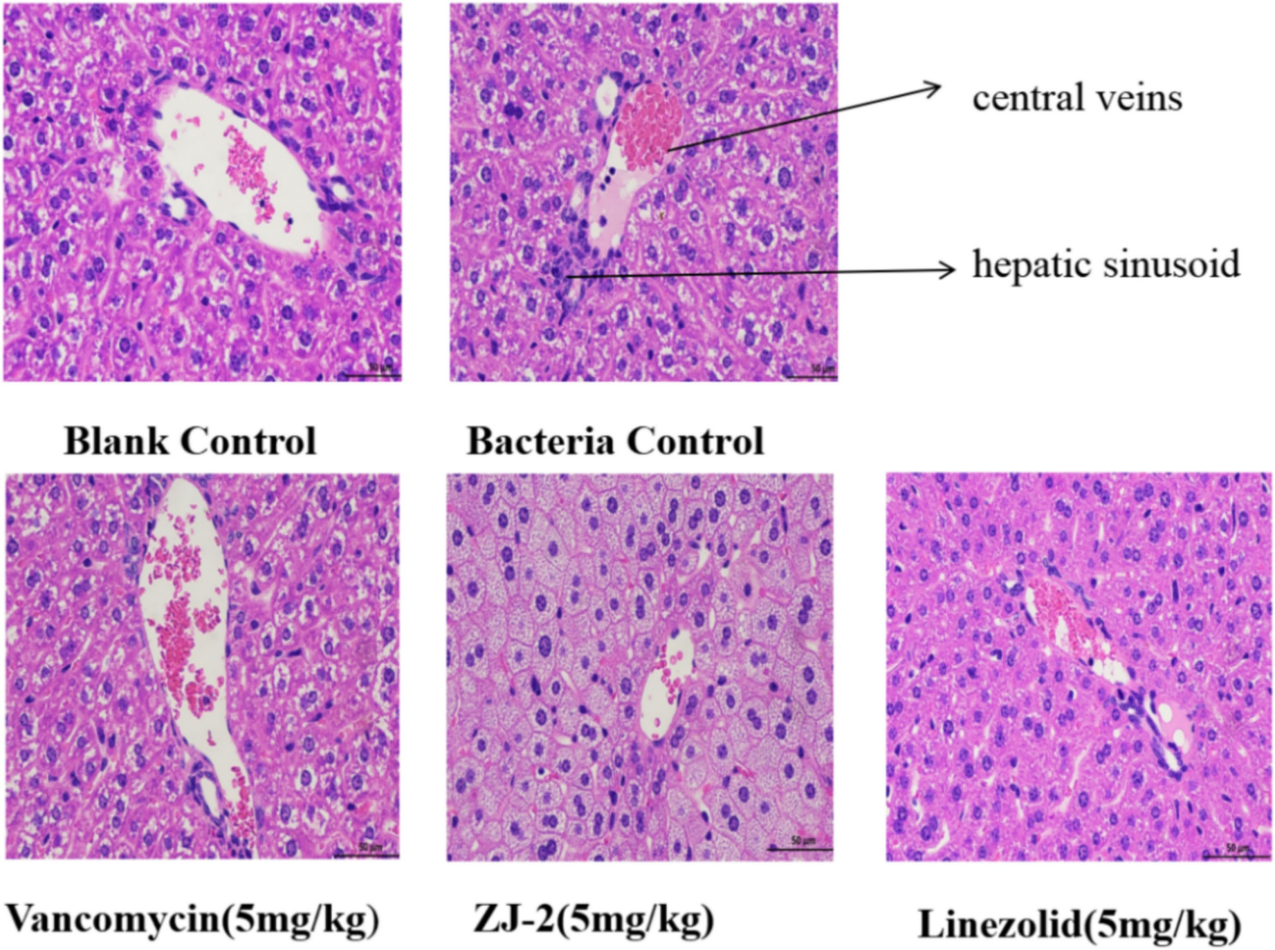
Liver histology stained with H&E. The effect of **ZJ-2** on the pathological changes of liver tissue was caused by *E. faecium* E3101. HE staining’s view is 50 μm.

The liver tissues were embedded in paraffin and stained with H&E for histological examination. As shown in Figure 10, HE staining revealed a significant decrease in inflammatory cell infiltration in ZJ-2-treated group compared with the *E. faecium* E3101 infected group. The nucleus was blue, and the cytoplasm and extracellular matrix were pink in different shades. The analysis of the liver histology is shown in Figure 10. The bacterial control group showed micro-and macro-vesicular fat deposition, lobular inflammatory cell infiltrate, and hepatocellular ballooning. The ZJ-2-treated group had significantly decreased fat deposition and improved the necrosis of liver tissue and inflammatory infiltrating cells caused by *E. faecium* E3101 in model mice. The results showed that ZJ-2 was more effective than LZD against *E. faecium* E3101.

## Discussion

With the increase in enterococci, multidrug-resistant biofilm-associated infection is becoming a growing problem worldwide (Mascio et al., 2014; Dai et al., 2022). This requires a deeper understanding of how enterococci biofilm develops, and how antibiotic resistance transfer takes place in these biofilms. Biofilms are defined as structured ecosystems in which microbes are attached to surfaces and embedded in a matrix composed of polysaccharides, extracellular DNA (eDNA) and proteins (Yu et al., 2019).

The best-characterized matrix component of *E. faecium* biofilm is eDNA (Thomas et al., 2009). Enzymatic degradation of eDNA can prevent, disperse, or sensitize biofilm to antimicrobials, and its release from cells is dependent on the hydrolases SagA and autolysin AtlA, which mediate lysis of bacterial cells (Yu et al., 2019). **ZJ-2** may reduced eDNA release and biofilm formation through in down-regulating the gene expression of peptidoglycan hydrolase, which was consistent with previous findings that autolysin AtlA is responsible for eDNA release in *E. faecium* biofilms. Furthermore, **ZJ-2** can be docked into SagA protein, which implied the antibacterial effect against *E. faecium* by inhibiting bacterial growth and daughter cell separation.

In addition, biofilms can shield bacteria from immune detection or phagocytosis, serving as an effective mechanism of immune evasion (Dai et al., 2022). *E. faecium* biofilm induces expression of proinflammatory cytokines (TNF-α and IL-6) and promotes survival within dendritic cells and macrophages (Supplementary Figure S2), which further enhanced persistence in the host (Todoriki et al., 2001). Aggregation substance, a surface protein encoded on the pheromone-inducible plasmids of enterococci, has been shown to increase adherence. Enterococcal surface protein, Esp., enhances biofilm formation by *E. faecalis*. Both contribute to virulence, the degradation of host tissues, and biofilm formation (Ali et al., 2021). **ZJ-2** had effectively down-regulated adherence-related genes, and reduced biofilm formation.

In conclusion, this diarylurea scaffold was found as anti-multidrug-resistant enterococci agents. **ZJ-2** showed benign drug-like property. Our study provided mechanism evidence of this diarylurea molecule with potent antibacterial and anti-biofilm activities through regulating the expression of peptidoglycan hydrolase genes and adherence-related genes against *E. faecium*. Inhibition of this process in the clinical isolates changed their inflammatory response and bacterial growth, adhesion, aggregation, biofilm formation and finally pathogenicity. Discovering novel anti-enterococci scaffolds targeting peptidoglycan hydrolases may be a successful therapeutic strategy for fighting life-threatening enterococci diseases.

## Data availability statement

The original contributions presented in the study are included in the article/Supplementary material, further inquiries can be directed to the corresponding author/s.

## Ethics statement

The animal study was reviewed and approved by the Ethics Committee of Anhui University of Science and Technology, China (IEC No: 201902).

## Author contributions

All authors listed have made a substantial, direct, and intellectual contribution to the work and approved it for publication.

## Funding

Financial support was provided by 2021 Scientific Research Project of Anhui Provincial Health Commission (AHWJ2021b054) and Anhui Provincial Natural Science Foundation (nos. 2008085MH261 and 2208085QH282).

## Conflict of interest

The authors declare that the research was conducted in the absence of any commercial or financial relationships that could be construed as a potential conflict of interest.

## Publisher’s note

All claims expressed in this article are solely those of the authors and do not necessarily represent those of their affiliated organizations, or those of the publisher, the editors and the reviewers. Any product that may be evaluated in this article, or claim that may be made by its manufacturer, is not guaranteed or endorsed by the publisher.

## Supplementary material

The Supplementary material for this article can be found online at: https://www.frontiersin.org/articles/10.3389/fmicb.2022.1071255/full#supplementary-material
