# Supplementary material for: Antibacterial and anti-biofilm activity of diarylureas against Enterococcus faecium by suppressing the gene expression of peptidoglycan hydrolases and adherence
Source: Front Microbiol. 2022 Dec 15;13:1071255. doi: 10.3389/fmicb.2022.1071255 (PMC9797508; doi:10.3389/fmicb.2022.1071255)
Supplement: Supplementary file 1 [file Data_Sheet_1.PDF]

Supporting information

Antibacterial and anti-biofilm activity of diarylureas against *Enterococcus faecium* by suppressing the gene expression of peptidoglycan hydrolases and adherence

Yunfeng Xie[1,†], Lei Wang[2,†], Yang Yang[1], Liang Zha[3], Jiazhen Zhang[1], Kuanrong Rong[3], Wenjian Tang[3*] and Jing Zhang[2*]

[1] School of Medicine, Anhui University of Science and Technology, Huainan 232001, China

[2] Anhui Prevention and Treatment Center for Occupational Disease, Anhui No. 2 Provincial People's Hospital, Hefei 230022, China

[3] School of Pharmacy, Anhui Medical University, Hefei 230032, China

Content

General procedure for the preparation of compounds **ZJ-1~ZJ-12**……………….**S2~S4**

FIGURE S1. The chemical structures of compound **ZJ-1~ZJ-12**………………….**S5**

TABLE S1. qRT-PCR primers in this study……….....................………….………**S6**

TABLE S2. MIC of compounds **ZJ-1~12** ………………...……….……....................**S7**

TABLE S3. MIC of active compounds against MRSA………………...……..……**S8**

FIGURE S2. Inhibition of *E. faecium*-induced IL-6 and TNF-α………...……….**S9**

General procedure for the preparation of compounds ZJ-1~12

A series of diphenylureas were synthesized from the condensation of substituted anilines with triphosgene in basic dichloromethane. Substituted arylamine **SM-1** (1.0 mmol) was added to a solution of triphosgene (0.33 mmol) in basic dichloromethane (DCM: 7 mL; $NaHCO_3$: 1 mL), then the reaction mixture was stirred under an ice bath for 15 min, the aryl isocyanates (**1**) were obtained. Substituted arylamine **SM-2** (1.0 mmol) was added into above reaction solution and the reaction was stirred at room temperature overnight (monitored by TLC) (Scheme S1). The mixture was extracted with dichloromethane (25 mL × 3), dried over anhydrous Na_2SO_4, filtered and concentrated in vacuo. The residue was purified by silica gel column chromatography (petroleum ether/EtOAc, 4: 1 to 2: 1) to give the title product **ZJ-1~12** (Figure S1).

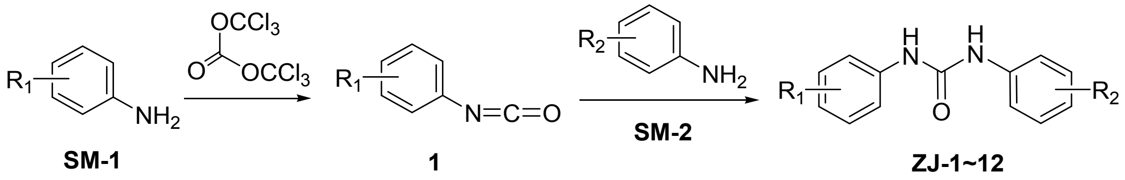

Scheme S1. Synthesis of compounds **ZJ-1~ZJ-12**.

Compound ZJ-1: 1,3-bisphenylurea

White powder, yield 92%; m.p. 235–238 °C; TOF-HRMS: *m/z* $[M + H]^+$ calcd for $C_{13}H_{12}N_2O$: 212.2472; found: 272.2466.

Compound ZJ-2: 1,3-bis(3-(trifluoromethyl)phenyl)urea

White powder, yield 95%; m.p. 189–191 °C; ^1H NMR (400 MHz, DMSO-d_6) δ 9.25 (s, 2H), 7.66 (m, 8H); ^{13}C NMR (126 MHz, DMSO-d_6) δ 152.6, 143.6 (2C), 126.5 (q, J = 3.8 Hz, 4C), 125.0 (d, J = 271.2 Hz, 2C), 122.6 (q, J = 32.1 Hz, 2C), 118.6 (4C); TOF-HRMS: *m/z* $[M + H]^+$ calcd for $C_{15}H_{10}F_6N_2O$: 349.0791; found: 349.0790.

Compound ZJ-3: 1,3-bis(3-(trifluoromethyl)phenyl)urea

White powder, yield 95%; m.p. 200–202 °C; ^1H NMR (500 MHz, DMSO-d_6) δ 9.18 (s, 2H), 8.02 (d, J = 2.1 Hz, 2H), 7.62 (d, J = 8.2 Hz, 2H), 7.53 (t, J = 7.9 Hz,

2H), 7.34 (d, *J* = 7.6 Hz, 2H); ^{13}C NMR (126 MHz, DMSO-d_6) δ 153.0, 140.8 (2C), 130.4 (2C), 130.0 (d, *J* = 31.4 Hz, 2C), 125.7 (t, *J* = 272.0 Hz, 2C), 122.61 (2C), 118.9 (d, *J* = 4.1 Hz, 2C), 115.0 (2C). TOF-HRMS: *m/z* [M + H]$^+$ calcd for $C_{15}H_{10}F_6N_2O$: 349.0791; found: 349.0792.

Compound ZJ-4: 1,3-bis(2-(trifluoromethyl)phenyl)urea

White powder, yield 92%; m.p. 200–202 °C; ^{1}H NMR (400 MHz, DMSO-d_6) δ 8.74 (s, 2H), 7.83 (d, *J* = 8.2 Hz, 2H), 7.73-7.60 (m, 4H), 7.32 (t, *J* = 7.6 Hz, 2H); TOF-HRMS: *m/z* [M + H]$^+$ calcd for $C_{15}H_{10}F_6N_2O$: 349.0791; found: 349.0790.

Compound ZJ-5: 1,3-bis(4-fluorophenyl)urea

Yellow powder, yield 96%; m.p. 220–222 °C; ^{1}H NMR (400 MHz, DMSO-d_6) δ 8.98 (s, 2H), 7.71 (t, *J* = 2.0 Hz, 2H), 7.42-7.16 (m, 4H), 7.04 (m, 2H); TOF-HRMS: *m/z* [M + H]$^+$ calcd for $C_{13}H_{10}F_2N_2O$: 249.0844; found: 249.0844.

Compound ZJ-6: 1,3-bis(3-fluorophenyl)urea

Yellow powder, yield 95%; m.p. 215–217 °C; ^{1}H NMR (400 MHz, DMSO-d_6) δ 8.98 (s, 2H), 7.50 (m, 2H), 7.32 (m, 2H), 7.14 (m, 2H), 6.80 (m, 2H); TOF-HRMS: *m/z* [M + H]$^+$ calcd for $C_{13}H_{10}F_2N_2O$: 249.0844; found: 249.0844.

***C*ompound ZJ-7: 1,3-bis(2-fluorophenyl)urea**

Yellow powder, yield 94%; m.p. 221–223 °C; 1H NMR (400 MHz, DMSO-d6) δ 9.12 -8.88 (m, 2H), 8.19 (m, 2H), 7.25 (m, 2H), 7.15 (m, 2H), 7.02 (m, 2H); TOF-HRMS: m/z [M + H]+ calcd for C13H10F2N2O: 249.0844; found: 249.0843.

Compound ZJ-8: 1-(4-fluorophenyl)-3-(4-(trifluoromethyl)phenyl)urea

Yellow powder, yield 93%; m.p. 195–197 °C; 1H NMR (400 MHz, DMSO-d6) δ 9.10 (s, 1H), 8.84 (s, 1H), 7.65 (m, 2H), 7.47 (m, 4H), 7.13 (m, 2H); ^{13}C NMR (126 MHz, DMSO-d_6) δ 158.1 (d, *J* = 251.8 Hz, 1C), 152.9, 143.9, 136.1 (d, *J* = 2.4 Hz, 1C), 126.5 (q, *J* = 3.6 Hz, 2C), 125.0 (d, *J* = 271.1 Hz, 1C), 121.5 (2C), 120.8 (d, *J* =

7.7 Hz, 1C), 118.3 (2C), 115.8 (dd, J = 22.3, 9.8 Hz, 2C); TOF-HRMS: *m/z* $[M + H]^+$ calcd for $C_{14}H_{10}F_4N_2O$: 299.0812; found :299.0812.

Compound ZJ-9: 1,3-bis(4-methoxyphenyl)urea

Brown powder, yield 93%; m.p. 198–200 °C; ^1H NMR (400 MHz, DMSO-d_6) δ 8.37 (s, 2H), 7.34 (m, 4H), 6.86 (m, 4H), 3.71 (s, 6H); TOF-HRMS: *m/z* $[M + H]^+$ calcd for $C_{15}H_{16}N_2O3$: 273.1234; found: 273.1233.

Compound ZJ-10: 1,3-bis(3,4-difluorophenyl)urea

White powder, yield 95%; m.p. 223–225 °C; ^1H NMR (500 MHz, DMSO-d_6) δ 8.96 (s, 2H), 7.65 (m, 2H), 7.35 (m, 2H), 7.15 (m, 2H); ^{13}C NMR (126 MHz, DMSO-d_6) δ 152.9, 149.6 (dd, J = 242.6, 13.2 Hz, 2C), 145.1 (dd, J = 239.9, 12.8 Hz, 2C), 137.0 (dd, J = 9.6, 2.5 Hz, 2C), 117.8 (2C), 115.1 (2C), 107.8 (2C); TOF-HRMS: *m/z* $[M + H]^+$ calcd for $C_{13}H_8F_4N_2O$: 285.0646; found: 285.0646.

Compound ZJ-11: 1,3-bis(3-chlorophenyl)urea

White powder, yield 94%; m.p. 202–204 °C; ^1H NMR (400 MHz, DMSO-d_6) δ 8.97 (s, 2H), 7.71 (t, J = 2.0 Hz, 2H), 7.28 (m, 4H), 7.04 (m, 2H); TOF-HRMS: *m/z* $[M + H]^+$ calcd for $C_{13}H_{10}Cl_2N_2O$: 281.0265; found: 281.0263.

Compound ZJ-12: 1,3-bis(6-chloropyridin-2-yl)urea

Yellow powder, yield 94%; m.p. 214–216 °C; ^1H NMR (400 MHz, DMSO-d_6) δ 9.90 (s, 2H), 7.84 (m, 4H), 7.14 (d, J = 7.3 Hz, 2H); TOF-HRMS: *m/z* $[M + H]^+$ calcd for $C_{11}H_8Cl_2N_4O$: 283.0160; found: 283.0159.

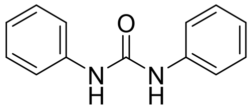

ZJ-1

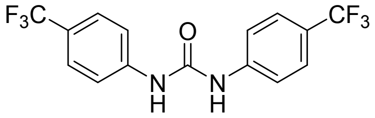

ZJ-2

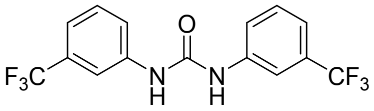

ZJ-3

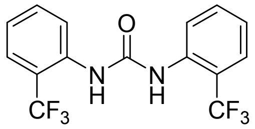

ZJ-4

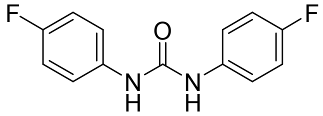

ZJ-5

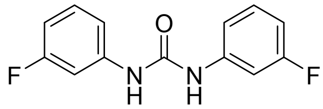

ZJ-6

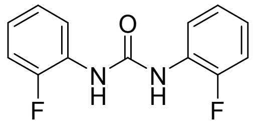

ZJ-7

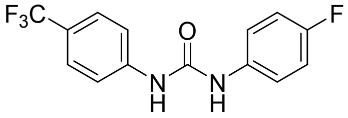

ZJ-8

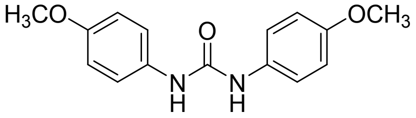

ZJ-9

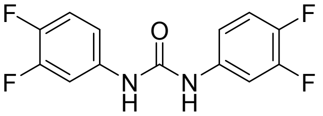

ZJ-10

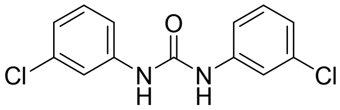

ZJ-11

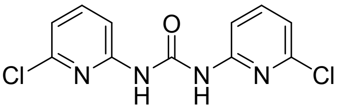

ZJ-12

FIGURE S1. The chemical structures of compound **ZJ-1~ZJ-12**.

TABLE S1. qRT-PCR primers in this study

Primer	Sequence	Reference
RT-*agg*-F	TCTTGGACACGACCCATGAT	Hashem et al., 2017
RT-*agg*-R	AGAAAGAACATCACCACGAGC	Hashem et al., 2017
RT-*esp*-F	AATTGATTCTTTAGCATCTGG	Zheng et al., 2017
RT-*esp*-R	AGATTCATCTTTGATTCTTGG	Zheng et al., 2017
RT-*atlA*-F	AACAGCACCAACGGATTAC	Dubrac et al., 2007
RT-*atlA*-R	CATAGTCAGCATAGTTATTCATTG	Dubrac et al., 2007
RT-*sagA*-F	ATGACCTTGACTGCCGTAGCAT	Paganelli et al., 2015
RT-*sagA*-R	TTACATGCTGACAGCAAAGTCAG	Paganelli et al., 2015
gyrB-F	GGTGCTGGGCAAATACAAGT	Valliammai et al., 2019
gyrB-R	TCCCACACTAAATGGTGCAA	Valliammai et al., 2019

TABLE S2 MIC (μmol/L) of compounds **ZJ-1**~ **ZJ-12** against strains. [1]

Compound	Gram-positive			Gram-negative		fungus
	SA	MRSA	EF	EC	PA	CA
**ZJ-1**	> 200	> 200	>200	> 200	> 200	> 200
**ZJ-2**	0.19	0.39	0.78	> 200	> 200	> 200
**ZJ-3**	0.39	0.39	6.25	> 200	> 200	> 200
**ZJ-4**	> 200	> 200	>200	> 200	> 200	> 200
**ZJ-5**	>200	>200	>200	> 200	> 200	> 200
**ZJ-6**	> 200	> 200	>200	> 200	> 200	> 200
**ZJ-7**	> 200	> 200	>200	> 200	> 200	> 200
**ZJ-8**	0.78	1.56	6.25	> 200	> 200	> 200
**ZJ-9**	100	100	100	200	200	200
**ZJ-10**	1.56	3.125	3.125	> 200	> 200	> 200
**ZJ-11**	> 200	> 200	>200	> 200	> 200	> 200
**ZJ-12**	> 200	> 200	>200	> 200	> 200	> 200
cefoxitin	25	100	50	50	> 200	nd [2]
ofloxacin	0.78	1.56	1.56	0.39	1.56	nd
linezolid	3.125	6.25	3.125	> 50	> 50	nd

MIC representing mean values of at least three replicates;

[1] SA (ATCC6538): *Staphylococcus aureus*, MRSA: Methicillin-resistant *S. aureus* (ATCC2858): clinical resistance to clindamycin, erythromycin, gentamicin and levofloxacin, EF: *Enterococcus faecium*; EC: *Escherichia coli*, PA: *Pseudomonas aeruginosa*, CA: *Candida albican* (ATCC10231);

[2] nd: no detected.

TABLE S3 MIC (µmol/L) of active compounds against isolated clinical MRSA strains from different specimen.

**compound**	MIC (µmol/L)			
	MRSA2858	MRSA0101	MRSA05107	MRSA10105
**ZJ-2**	0.39	0.78	1.56	0.78
**ZJ-3**	0.39	1.56	3.125	1.56
**ZJ-8**	1.56	3.125	6.25	6.25
Vancomycin[1]	0.78	0.78	1.56	1.56

MIC representing mean values of at least three replicates;

[1] It is vancomycin HCl (Solarbio®, Lot. No. 1115F021).

Inhibition of *E. faecium*-induced IL-6 and TNF-α in Caco-2 cells

The post confluent Caco-2 cells were further incubated for 18h in serum-free media (Thermo Fisher Co.). The experiment was divided into three groups: control group, **ZJ-2** MIC group and **ZJ-2** 2MIC group. And subsequently, cells were treated with *E. faecium* E 3101 with or without compound **ZJ-2** at 37 °C for 24 h. The culture supernatants were collected, and the levels of Interleukin 6 (IL-6) and tumor necrosis factor-α (TNF-α) in the culture supernatants were determined using an ELISA kit (R&D Systems) in Caco-2 cells. Samples were tested in triplicate.

ZJ-2 inhibits IL-6 and TNF-α production in intestinal epithelial cells

The Caco-2 cell line was well established and was commonly used to study the effects of Enterococci on intestinal epithelial cells.The proinflammatory cytokine response in the gastrointestinal tract is influenced by the gut microbiota. Among them, the cytokine expression in intestinal epithelial cells can be modulated by enterococci. Alations in cytokine release can in turn regulate epithelial cell function.

The inflammatory factors of IL-6 and TNF-α in MIC and 2MIC **ZJ-2** treatment groups were significantly decreased compared with control group. The results showed that the level of IL-6 and TNF-α were significantly decreased by 29.0%, 44.8% and 11%, 24.7%, respectively. Compound **ZJ-2** decreased the level of IL-6 and TNF-α in a dose-dependent manner (Figure S2). These studies suggested that compound **ZJ-2** had inhibited inflammatory responses of intestinal epithelial cells *in vitro*.

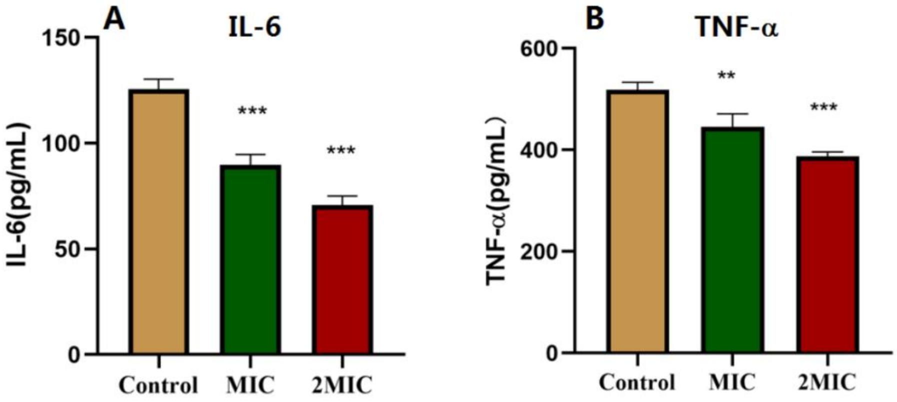

FIGURE S2. Inhibition of *E. faecium* induced IL-6 and TNF-α production in Caco-2 cells. The level of IL-6 (**A**) and TNF-α (**B**) were expressed as mean-standard deviation from triplicate samples. Statistical significance compared with control is indicated by $^{**}p < 0.05$ and $^{***}p < 0.01$.

References

Dubrac, S., Boneca, I. G., Poupel, O., & Msadek, T. (2007). New insights into the WalK/WalR (YycG/YycF) essential signal transduction pathway reveal a major role in controlling cell wall metabolism and biofilm formation in *Staphylococcus aureus*. *J. Bacteriol*. 189, 8257–8269. doi: 10.1128/JB.00645-07

Hashem, Y. A., Amin, H. M., Essam, T. M., Yassin, A. S., & Aziz, R. K. (2017). Biofilm formation in enterococci: genotype-phenotype correlations and inhibition by vancomycin. *Sci. Rep*. 7, 5733. doi: 10.1038/s41598-017-05901-0

Paganelli, F. L., de Been, M., Braat, J. C., Hoogenboezem, T., Vink, C., Bayjanov, J., et al. (2015). Distinct SagA from Hospital-Associated Clade A1 *Enterococcus faecium* Strains Contributes to Biofilm Formation. *Appl. Environ. Microb*. 81, 6873–6882. doi:10.1128/AEM.01716-15

Valliammai, A., Sethupathy, S., Priya, A., Selvaraj, A., Bhaskar, J. P., Krishnan,V., & Pandian, S. K. (2019). 5-Dodecanolide interferes with biofilm formation and reduces the virulence of Methicillin-resistant *Staphylococcus aureus* (MRSA) through up regulation of agr system. *Sci. Rep.* 9, 13744. doi: 10.1038/s41598-019-50207-y

Zheng, J. X., Wu, Y., Lin, Z. W., Pu, Z. Y., Yao, W. M., Chen, Z., Li, et al. (2017). Characteristics of and Virulence Factors Associated with Biofilm Formation in Clinical *Enterococcus faecalis* Isolates in China. *Front. Microbiol.* 8, 2338. doi: 10.3389/fmicb.2017.02338
